# Early-Stage Alzheimer's Disease Affects Fast But Not Slow Adaptive Processes in Motor Learning

**DOI:** 10.1523/ENEURO.0108-24.2024

**Published:** 2024-06-07

**Authors:** Katrin Sutter, Leonie Oostwoud Wijdenes, Robert J. van Beers, Jurgen A. H. R. Claassen, Roy P. C. Kessels, W. Pieter Medendorp

**Affiliations:** ^1^Donders Institute for Brain, Cognition and Behaviour, Radboud University, Nijmegen 6525 GD, The Netherlands; ^2^Department of Human Movement Sciences, Vrije Universiteit Amsterdam, Amsterdam 1081 BT, The Netherlands; ^3^Department of Geriatric Medicine, Radboud University Medical Center, Nijmegen 6525 GA, The Netherlands; ^4^Department of Medical Psychology and Radboudumc Alzheimer Center, Radboud University Medical Center, Nijmegen 6525 GA, The Netherlands; ^5^Vincent van Gogh Institute for Psychiatry, Venray 5803 DM, The Netherlands

**Keywords:** declarative memory, dual-rate learning, force-field adaptation, hierarchical Bayesian modeling, reaching

## Abstract

Alzheimer's disease (AD) is characterized by an initial decline in declarative memory, while nondeclarative memory processing remains relatively intact. Error-based motor adaptation is traditionally seen as a form of nondeclarative memory, but recent findings suggest that it involves both fast, declarative, and slow, nondeclarative adaptive processes. If the declarative memory system shares resources with the fast process in motor adaptation, it can be hypothesized that the fast, but not the slow, process is disturbed in AD patients. To test this, we studied 20 early-stage AD patients and 21 age-matched controls of both sexes using a reach adaptation paradigm that relies on spontaneous recovery after sequential exposure to opposing force fields. Adaptation was measured using error clamps and expressed as an adaptation index (AI). Although patients with AD showed slightly lower adaptation to the force field than the controls, both groups demonstrated effects of spontaneous recovery. The time course of the AI was fitted by a hierarchical Bayesian two-state model in which each dynamic state is characterized by a retention and learning rate. Compared to controls, the retention rate of the fast process was the only parameter that was significantly different (lower) in the AD patients, confirming that the memory of the declarative, fast process is disturbed by AD. The slow adaptive process was virtually unaffected. Since the slow process learns only weakly from an error, our results provide neurocomputational evidence for the clinical practice of errorless learning of everyday tasks in people with dementia.

## Significance Statement

Experimental and modeling studies of motor adaptation suggest a dual-rate process of motor memory formation. We examined force-field motor adaptation in patients diagnosed with impaired declarative memory due to early-stage Alzheimer's disease. Using hierarchical Bayesian modeling, we show that their fast, but not slow, process of adaptation is affected, suggesting that the fast process in motor adaptation shares resources with the declarative memory system.

## Introduction

Impaired declarative memory is a hallmark of Alzheimer's disease (AD), the most common cause of dementia. While patient brains show widespread neurodegeneration, this progressive memory loss is strongly related to bilateral atrophy of the medial temporal lobe, including the entorhinal cortex and the hippocampus proper, as well as atrophy in parietal areas (Hyman et al., 1984; Braak and Braak, 1991, 1996; Van Hoesen et al., 1991; Foundas et al., 1997). Despite this loss, there is evidence that aspects of learning and memory that rely more on automatic and unconscious processing, referred to as nondeclarative or procedural memory, are relatively intact (Shadmehr et al., 1998; Zanetti et al., 2001; van Halteren-van Tilborg et al., 2007; Kessels et al., 2011; De Wit et al., 2021, 2022).

Motor learning has traditionally been regarded as a form of nondeclarative memory. It is defined as the process of (re)gaining or retaining a given level of motor performance (Krakauer et al., 2019). Indeed, AD patients are still able to (re)learn motor tasks, although learning success depends on the type of the task (Willingham et al., 1997; van Tilborg and Hulstijn, 2010), how feedback is provided (van Halteren-van Tilborg et al., 2007), whether rewards are present or not (Wong et al., 2019), and how engaged the patient is in the task (Laffan et al., 2010).

More recently, it has been suggested that motor learning not only relies on nondeclarative memory but also involves declarative processing (Mazzoni and Krakauer, 2006; Keisler and Shadmehr, 2010; Taylor and Ivry, 2011; Taylor et al., 2014; McDougle et al., 2022).

The arguments find their basis in a computational theory of error-based motor learning. This theory proposes that the learning process involved in acquiring a new mapping between motor commands and behavioral outcome involves a fast adaptive process that learns quickly but also decays rapidly and a slow process that learns slowly but has good retention (M. A. Smith et al., 2006). Behavioral and neural evidence for such dual-rate learning has been reported for both force-field and visuomotor adaptation using spontaneous recovery paradigms (Lee and Schweighofer, 2009; Trewartha et al., 2014; Inoue et al., 2015; Sarwary et al., 2018).

Because the fast process is mainly driven by large movement errors, Keisler and Shadmehr (2010) argued that it shares resources with the declarative memory process. They reasoned that large movement errors enter the learner's awareness and are thus explicitly processed (Malfait and Ostry, 2004) and hence engage the declarative memory system. In support, the authors showed that performing a declarative memory task (word-list recall) after completion of a reach adaptation task produced interference with the memory of the fast process, but not the slow process (Keisler and Shadmehr, 2010). Taylor et al. (2014) showed that implicit adaptation demonstrated slow adaptation dynamics, while explicit adaptation demonstrated fast adaptation dynamics. Finally, it has been shown that limiting the movement preparation time suppresses the recruitment of explicit processing, such that learning is best described by a single implicit process (Fernandez-Ruiz et al., 2011; Haith et al., 2015; Leow et al., 2017).

Therefore, if explicit, declarative processing affects the ability of the fast process to lay down motor memories, it can be hypothesized that its retention rate is lower in AD patients than in controls. Here, after careful neuropsychological examination, we tested 20 early-stage AD patients in a spontaneous recovery paradigm using a force-field adaptation task with reaching movements. Compared with age- and education level-matched healthy control participants, their data show that the fast, but not the slow, adaptive process is affected, which not only refines computational theories of motor learning but also allows for possible clinical translation.

## Materials and Methods

### Participants

The Medical Ethics Committee of the Radboud University Medical Center judged this study exempt from formal medical ethical approval according to the WMO Act (CMO Arnhem-Nijmegen 2017-3162) due to the minimal burden it imposed on participants. The present study was subsequently approved by the ethics committee of the Social Sciences Faculty of Radboud University (ECG2017-0805-504). All participants (patients and controls) gave written consent to participate in the study and were reimbursed for their time at a rate of 10€ per hour.

All participants had normal or corrected-to-normal vision. The Edinburgh Handedness Inventory (Oldfield, 1971) showed that all but one participant (from the control group) were right-handed. Participants indicated their education level based on the Dutch educational system (range, 1–7, with 1, less than primary school; 7, academic degree; Verhage, 1964).

Patients were recruited from the memory clinic of the Radboud University Medical Center in the period between May 2017 and May 2019. Inclusion criterion for the patients was having a declarative memory impairment verified by neuropsychological assessment due to AD [either amnestic mild cognitive impairment (MCI) or mild dementia]. The exclusion criteria were a history of other neurological diseases that affect the brain (stroke, Parkinson's disease, brain tumor), a history of or an active psychiatric disorder (including psychotic disorders or substance use disorders), and no command of Dutch language. The main experimental group consisted of 20 patients (5 women, aged 60–87 years) who were all diagnosed with (amnestic) MCI due to AD or a mild Alzheimer's dementia. MCI due to AD refers to the symptomatic predementia phase of AD. In MCI patients, the degree of cognitive impairment is not age-appropriate (G. E. Smith et al., 1996; M. S. Albert et al., 2011). Clinical diagnoses were established based on a multidisciplinary assessment at the memory clinic of the Radboud University Medical Center and were supported by a clinical interview with the patients and their informants, neuroradiological findings, neuropsychological assessment, and a review of the patients’ medical history, in accordance with current criteria (M. S. Albert et al., 2011; McKhann et al., 2011). The clinical dementia rating (Hughes et al., 1982) of the patients was 0.5 (MCI) or 1 (mild dementia).

As a control group, we recruited 25 age- and education level-matched, cognitively unimpaired participants (Verhage, 1964). Four participants from this group were excluded from the analysis: one due to failure to follow task instructions, one due to experienced pain in the right arm when holding the robot handle, and two due to failure in experimental recording; hence 21 healthy participants (13 women, aged 61–87 years) were included in the analysis. This group was both age-matched (*t*_(39)_ = 0.5; *p *= 0.6; *t* test) and matched at the education level (*t*_(30.31)_ = 1.2; *p *= 0.26; *t* test). Visual inspection of the data of the left-handed participant, who performed the task with the right hand, did not reveal differences from right-handed participants’ data and therefore was included in the analysis. The demographic (and neuropsychological) characteristics of the AD patients and the control group are presented in Table 1.

**Table 1.** Demographic and neuropsychological characteristics of the AD patients and the control participants

	AD patients	Control participants
Age (years)	75.6 (6.5)	74.4 (7.4)
Number of men/women	15/5	9/12
Education level (1–7)	5.0 (1.9)	5.6 (1.1)
Education in years	13.7 (5.7)	15.6 (3.7)
Number of patients with CDR 0.5/1	6/14	n/a
MoCA total score (points)	20.1 (3.9)	26.4 (1.9)
MoCA-MIS (points)	3.5 (3.5)	12.7 (1.9)
NART (IQ)	101.2 (15.0)	108.6 (7.4)
Doors test (points)	12.0 (3.9)	17.9 (3.2)

Data are reported as mean (SD) or number. CDR, clinical dementia rating, 0.5–1 indicates very mild to mild dementia. MoCA, Montreal Cognitive Assessment, maximum score 30, education adjusted (1 extra point for those with 12 or less years of education). MoCA-MIS, Montreal Cognitive Assessment Memory Index Score, maximum score 15. NART, National Adult Reading Test, intelligence quotient estimate, maximum score 130. Doors test, visual recognition memory task, maximum score 24. Education level: 1, <6 years elementary school; 2, 6 years elementary school; 3, >6 years elementary school; 4, vocational training; 5, community college; 6, advanced vocational training; 7, university degree.

### Neuropsychological testing

All participants performed a set of neuropsychological tests before the reaching task. First, participants performed the Dutch version of the National Adult Reading Test (NART) to estimate their premorbid level of intellectual functioning (Schmand et al., 1992). Second, the Montreal Cognitive Assessment (MoCA) with the cued-recall and multiple-choice memory test items (enabling calculating the Memory Index Score, MoCA-MIS) was administered to assess global cognitive functioning and memory (Nasreddine et al., 2005; Julayanont et al., 2014). Finally, participants completed the doors test (Parts 1 and 2), a visual recognition memory test from the doors and people test (Baddeley et al., 1994) to assess declarative memory. In this test, participants view photographs of doors which they must remember and later recognize from a selection of similar doors. The test has two parts, where Part 1 is easier since Part 2 uses more similar doors. The results of the neuropsychological characteristics of the patients and control participants groups are presented in Table 1.

An independent sample *t* test on the IQ estimates (based on the NART) did not reveal any significant differences between the two groups (*t*_(27.5)_ = 2.0; *p *= 0.057; *t* test). However, as expected, the AD patients had worse global cognitive functioning than the control group (MoCA, *t*_(27.2)_ = 6.6; *p *< 0.001; *t* test) and worse declarative memory performance (doors test, *t*_(39)_ = 5.3; *p *< 0.001; MoCA-MIS, *t*_(28.4)_ = −10.3; *p *< 0.001; *t* test).

### Experimental setup

**Figure 1.**
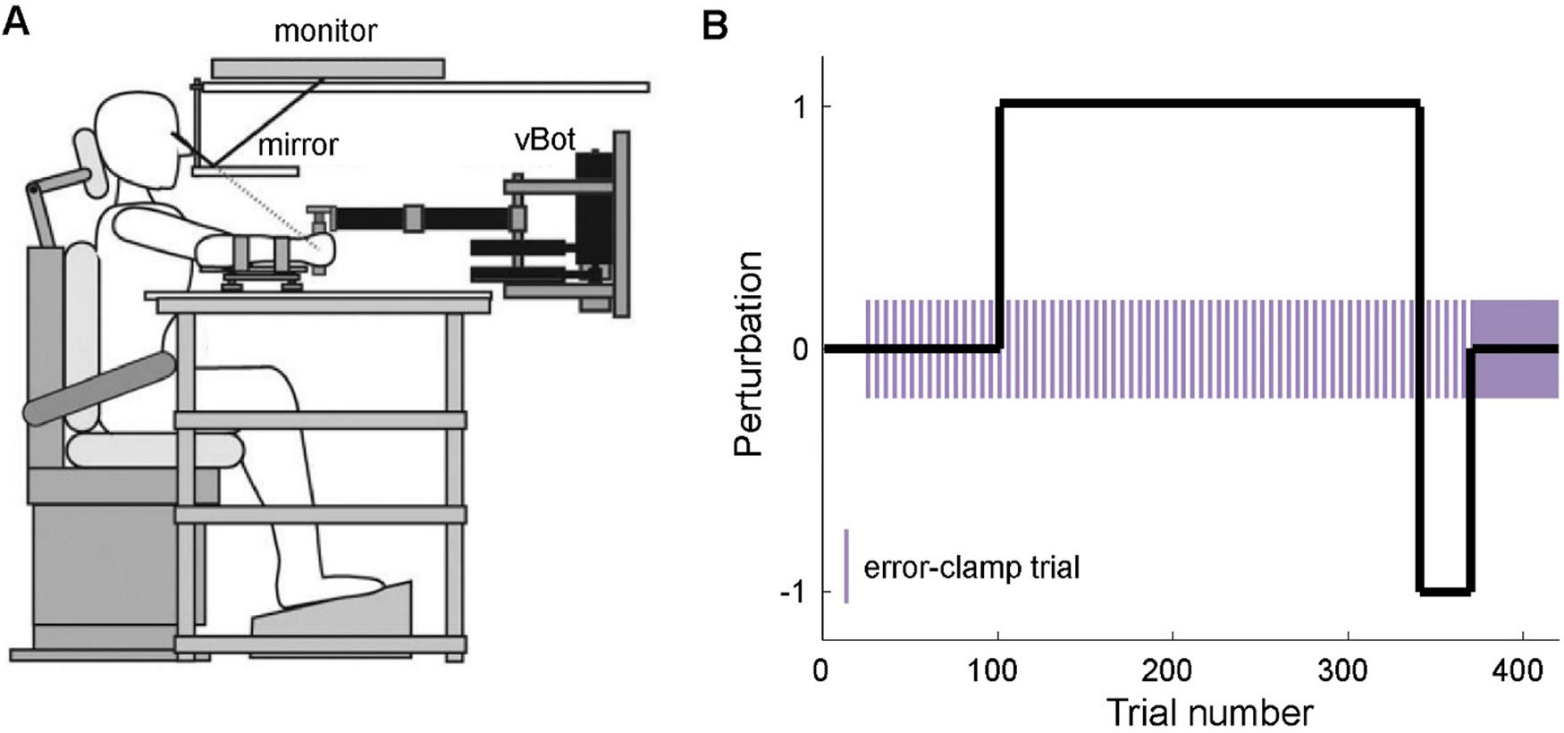
***A***, Experimental setup. Participants held the handle of a planar robotic manipulandum (vBot) with their right hand while their arm was resting on an air sled floating on a glass-top table. Visual stimuli were presented through a mirror. Image reproduced from Franklin and Wolpert (2008) with permission. ***B***, Experimental paradigm. The experiment started with 100 null-field trials; these were followed by 240 CW force-field trials, 30 CCW force-field trials, and finally 50 EC trials. To track the progression of learning, every fifth trial from trial 21 to 370 was an EC trial (purple bars).

During the experiment, participants sat in front of a planar robotic manipulandum (Howard et al., 2009). While holding the handle of the manipulandum, they performed reaching movements in the horizontal plane with their right arm. Participants did not have direct visual feedback of the arm due to a semisilvered mirror that covered the arm (Fig. 1*A*). An air sled underneath the right forearm allowed frictionless movements. All visual stimuli were presented on an LCD monitor (model VG278H, Asus) that was viewed via the semisilvered mirror. The refresh rate of the display was 120 Hz. Stimuli were shown in the same plane as the movements. Hand position, derived from the robot's handle position, was presented as a cursor (red disc, 0.35 cm radius). The target (yellow disc, 1 cm radius) was placed 12 cm out in the straight-ahead direction from the central home position (white disc, radius 1 cm). The home position was located ∼30 cm from the participant's chest. An auditory imperative stimulus and warning/error message were presented via speakers that were behind the workspace of the experiment. Robot handle position data were measured at 1,000 Hz.

There were three types of trials: (1) null-field trials (robot forces were turned off); (2) curl force-field trials, and (3) error-clamp (EC) trials. In curl force-field trials, the robot produced forces that were perpendicular to the movement direction and proportional to the reach speed as follows:

(1) $$\left[\begin{array}{c} F_{x}\\ F_{y} \end{array}\right]=b\left[\begin{array}{cc} 0 & 1\\ -1 & 0 \end{array}\right]\left[\begin{array}{c} v_{x}\\ v_{y} \end{array}\right],$$

where *x* and *y* are the lateral and sagittal directions, $F_{x}$ and $F_{y}$ are the robot forces applied at the hand, $v_{x}$ and $v_{y}$ are hand velocities, and b is the field constant (±13 Ns/m). The sign of the field constant determined the direction of the force field. ECs served to measure the adaptation index (AI). In EC trials, the hand was constrained to a straight path from the start to the target with a spring constant of 6,000 N/m and a damping constant of 7.5 Ns/m. At the end of each reach, participants were asked to relax their arm, while the arm was passively returned to the start position following a minimum jerk profile with a duration of 700 ms.

### Experimental paradigm

The reaching task was first explained to the participant by the experimenter using a paper version of the task workspace and a dummy robot handle at the same desk where the neuropsychological tests were administered. Once the participant understood the task, they were asked to take a seat at the desk where the reaching experiment took place. To start a trial, participants placed the cursor inside the home disc. After 500 ms, the yellow target appeared, which was accompanied by an auditory beep. Participants were instructed to move in a straight line to the target as soon as it appeared. The target turned green if the cursor was in the target. At the end of the reach, the robot returned the hand to the start position. The intertrial interval was set at 200 ms. In order to get accustomed with the experimental setup, participants performed 10 practice trials with the vBot before the experiment. If necessary, this practice block was repeated.

The experiment started with 100 null-field trials (Fig. 1*B*). Thereafter, the clockwise (CW) curl force field was turned on for 240 trials. This was followed by 30 trials of counterclockwise (CCW) curl force field, and finally participants performed 50 EC trials. From trial 21 to 370, the null-field and force-field trials were interleaved with EC trials (every fifth trial). In a debriefing after the experiment, none of the participant (patients or controls) had noticed the presence of clamp trials.

To ensure that participants did not slow down the reaching pace along the experiment, we have given a warning message “Move faster” at the end of the reach if movement duration (time between the movement onset and reach offset) was longer than 500 ms. A warning message “Stay in the target!” was displayed if the cursor left the 3-cm-radius area around the target disc within 200 ms after entering it. Warning messages did not lead to restarting or exclusion of the respective trial. Participants received an error message if they did not start the reaching movement within 1,000 ms and the trial was restarted. All warning and error messages were displayed for 1,250 ms.

### Data analysis

Analyses were performed using MATLAB 2018b (MathWorks). The reach onset was determined as the time point when the cursor speed exceeded 5 cm/s. The reach offset was determined as the time point 200 ms after the distance between the cursor and the center of the target disc was smaller than 3 cm. There were two exceptions to this criterion: (1) if the cursor after entering the 3 cm radius area around the target disc center left it again within 200 ms. In such cases, the reach offset was determined upon exiting the 3-cm-radius area; (2) if the cursor speed dropped below 5 cm/s outside the 3-cm-radius area from the target, the reach offset was determined at this timepoint.

Trials in which the participant moved <6 cm from the middle of the starting position were removed from further analysis. For each EC trial, we computed the AI, which represents the fraction of ideal force compensation in response to the curl force field. To this end, based on the velocity of the handle along the channel, we calculated the time-varying lateral force that would have been generated by the force field in a field trial. The force measured in the EC was regressed against this theoretical force, providing the regression coefficient, which was taken as the AI [see Joiner and Smith (2008) for more details]. Adaptation indices during the force-field trials were baseline corrected by subtracting the mean AI during the null trials in the beginning of the experiment.

### State–space modeling

We fitted a Bayesian hierarchical version of the dual-rate model (M. A. Smith et al., 2006) to the time course of the AI across the various phases of the experiment. According to this model, the total adaptation at trial n+1 can be represented by the sum of the states of a fast process $\left(x_{f}(n+1)\right)$ and a slow process $\left(x_{s}(n+1)\right)$ as follows:

(2) $$x_{f}(n+1)=R_{f}x_{f}(n)+\;L_{f}e(n)+\varepsilon_{f},$$

(3) $$x_{s}(n+1)=R_{s}x_{s}(n)+\;L_{s}e(n)+\varepsilon_{s},$$

(4) $$\varepsilon_{f}∼N(0,\sigma_{f}),$$

(5) $$\varepsilon_{s}∼N(0,\sigma_{s}).$$

The state of each process depends on the state at the previous trial n, multiplied by a retention rate (R), and the error of the previous trial, e(n), multiplied by the learning rate (L), while also Gaussian state noise ( $\varepsilon_{f}$, $\varepsilon_{s}$, Eqs. 4, 5) is added at each trial. Both processes have independent retention and learning rates, which were constrained as follows: $0<R_{f}\;<R_{s}<1$ and $0<L_{s}<L_{f}\;<1$.

The sum of the fast and the slow process is the net motor output (x(n+1)) as follows:

(6) $$x(n+1)=x_{f}(n+1)+x_{s}(n+1).$$

The actual movement output (y(n+1)) is equal to the sum of the net motor output (Eq. 6) and Gaussian output noise $\left(\varepsilon_{\mathrm{output}}\right)$ as follows:

(7) $$y(n+1)=x(n+1)+\varepsilon_{\mathrm{output}},$$

(8) $$\varepsilon_{\mathrm{output}}∼N(0,\sigma_{\mathrm{output}}).$$

The error (e(n)) on a trial (n) is defined as the difference between the actual movement output, y(n), and the applied perturbation force, f(n), as follows:

(9) e(n)=y(n)−f(n).

We used a hierarchical implementation of this dual-rate model, following Ferrea et al. (2022), to estimate the learning and retention rates of individual participants. The advantage of using hierarchical modeling is that the estimate of the individual parameters is informed by data from all other individuals in the same group (patients vs controls; Kruschke, 2015).

**Figure 2.**
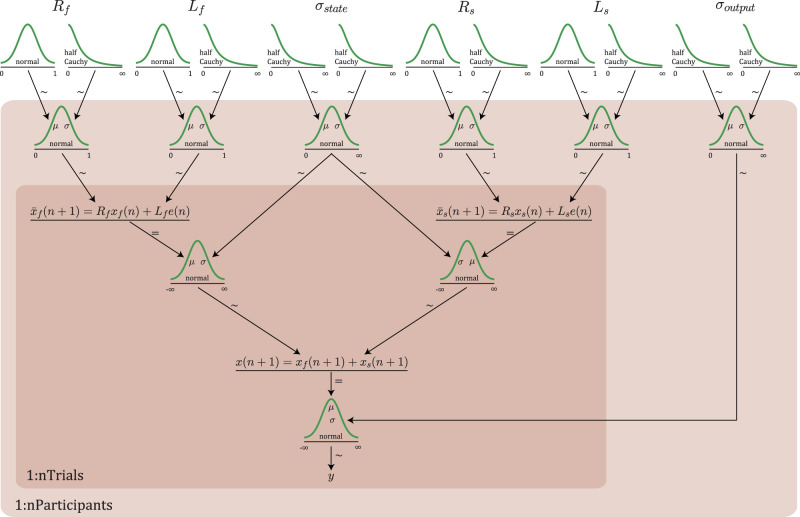
Diagram of the hierarchical implementation of the dual-rate model. Individual participant's learning and retention rate estimates for both the slow and fast process were sampled from truncated normal distributions (the priors shown at the second row), which were defined in terms of their mean μ and standard deviation σ. These parameters were hyperparameters that were sampled from the hyperpriors in the top row (truncated normal distributions for the means and half-Cauchy distributions for the standard deviations). The standard deviations σ of the fast and the slow state noise and the output noise were sampled from normal priors (in the second row). Their mean μ and standard deviation σ were sampled from half-Cauchy hyperpriors in the top row. The distributions on the white background were sampled at the group level; those on light red background at the participant level and those on the dark red background at the trial level.

Figure 2 shows a graphic diagram of the full hierarchical model. Each participant's learning and retention rates for both the slow and fast process were sampled from priors: normal distributions truncated to the interval [0, 1]. The means μ and standard deviations σ of these priors were hyperparameters of the hierarchical model, which were sampled from hyperpriors. For the mean μ, the hyperpriors were normal distributions truncated to the interval [0, 1]. The means of these hyperpriors were 0.998, 0.85, 0.1, and 0.1 for $R_{s}$, $R_{f}$, $L_{s}$, and $L_{f}$, respectively, and the standard deviations were 0.01, 0.5, 0.5, and 0.5 for these respective parameters. For the standard deviations of the priors, the hyperparameters were half-Cauchy distributions. For all learning and retention rates, these were Cauchy distributions with location parameter 0 and scale parameter 0.5, truncated to values >0. The standard deviation σ of both the fast and the slow state noise and the standard deviation of the output noise were constrained to values >0, and these were sampled from normal priors with hyperparameters mean μ and standard deviation σ. All these hyperparameters were sampled from half-Cauchy hyperpriors. For the means, the location and scale parameter were 0 and 1, respectively, whereas these were 0 and 0.5 for the standard deviations.

To have the parameter estimates mostly determined by the data, all hyperpriors were fairly uninformative, except for those pertaining to the means of the retention rates, which were chosen more informative to speed up the convergence of the Markov chains. We verified that making these hyperpriors less informative also led to the same parameter estimates but at the expense of a much lower effective sample size (ESS).

We used independent hierarchical models for the AD patients and for the healthy controls. Importantly, the same model with the same priors was used for both groups.

### Statistical analysis

Statistical analyses of the outcomes of the neuropsychological tests and the adaptation process with AIs were performed using SPSS Statistics 25. To compare the adaptation process along the reaching experiment, we compared the AIs of the AD patients and control participants in three different phases of the experiment: (1) at the plateau of adaptation during the CW force field (last 15 EC trials); (2) during CCW force field (last three EC trials); (3) and, finally, at the beginning of the final EC phase at the end of the experiment (first 12 EC trials). To this aim, we first calculated the mean AI for each phase per participant and compared groups with paired samples *t* tests.

Parameter estimation of the dual-rate model was conducted using Markov chain Monte Carlo (MCMC) methods in Stan (CmdStan version 2.17.1) via its MATLAB interface MatlabStan. The MCMC method gives representative samples of the posterior distribution of the model parameters given the data. We ran the model on four chains with a burn-in phase of 3,000 samples and 25,000 iterations for each chain. No thinning was used. We inspected the following MCMC diagnostics for each parameter: the convergence diagnostic $\hat{R}$, the ESS, and the Monte Carlo standard error (MCSE). For all hyperparameters, the fits revealed the $\hat{R}<1.022$, the ESS > 250, and the MCSE < 0.0023. Table 2 lists the $\hat{R}$, ESS, and MCSE for all hyperparameters.

**Table 2.** MCMC diagnostics of the model parameters

Parameter	AD patients			Control participants		
	ESS	$\hat{R}$	MCSE	ESS	$\hat{R}$	MCSE
$L_{f}-\mu$	1,668.1	1.0025	0.00031	2,538.0	1.0028	0.00008
$L_{f}-\sigma$	2,533.3	1.0017	0.00020	1,276.8	1.0025	0.00012
$L_{s}-\mu$	1,341.9	1.0017	0.00005	468.4	1.0131	0.00008
$L_{s}-\sigma$	2,467.0	1.0012	0.00003	432.8	1.0125	0.00009
$R_{f}-\mu$	562.6	1.0078	0.00228	650.4	1.0026	0.00037
$R_{f}-\sigma$	420.5	1.0102	0.00227	336.3	1.0165	0.00056
$R_{s}-\mu$	636.8	1.0054	0.00004	578.4	1.0039	0.00002
$R_{s}-\sigma$	3,013.7	1.0016	0.00001	874.7	1.0032	0.00001
$\sigma_{\mathrm{state}}-\mu$	432.9	1.0123	0.00022	536.1	1.0079	0.00015
$\sigma_{\mathrm{state}}-\sigma$	323.3	1.0153	0.00028	250.2	1.0214	0.00019
$\sigma_{\mathrm{output}}-\mu$	2,226.7	1.0026	0.00032	1,926.8	1.0016	0.00021
$\sigma_{\mathrm{output}}-\sigma$	2,767.8	1.0022	0.00024	2,238.6	1.0018	0.00016
$L_{f}*$	3,074.4	1.0015	0.00041	2,053.8	1.0018	0.00020
$L_{s}*$	4,569.4	1.0014	0.00007	1,184.7	1.0058	0.00010
$R_{f}*$	1,068.6	1.0044	0.00284	849.2	1.0053	0.00064
$R_{s}*$	1,403.7	1.0027	0.00004	1,319.4	1.0020	0.00002
$\sigma_{\mathrm{state}}*$	462.7	1.0137	0.00051	338.2	1.0209	0.00046
$\sigma_{\mathrm{output}}*$	1,008.5	1.0082	0.00072	791.9	1.0086	0.00055

Parameter names in the table correspond to the parameters estimated by the model. For parameters that were estimated for individual participants (noted with an asterisk), the mean of the participants is listed. ESS, effective sample size; $\hat{R}$, convergence diagnostic; MCSE, Monte Carlo standard error.

To validate our fitting procedure, we generated synthetic data from 20 artificial participants with parameters in the same range as estimated for our real participants. We then fit the model to these synthetic data in the same way as our actual data and determined how well the parameters of the artificial participants were recovered. Specifically, we determined the difference between the mean of the posterior of each parameter and the actual value used to generate the synthetic data. The mean absolute differences were *R_f_* < 0.03, *R_s_ *< 0.001, *L_f_ *< 0.013, *L_s_ *< 0.003, *σ*_state_ < 0.003, and *σ*_output_ < 0.008, demonstrating a successful recovery of the model.

We performed a posterior predictive check to assess whether the model gave valid predictions of the data. A grand-average visualization (Fig. 4) was generated from averaging the posterior predictive checks for all participants, separately for patients and healthy controls. Individual posterior predictions were generated from random draws (*n *= 500) of the posterior distributions of the estimated learning and retention rates of the fast and the slow process, while the state and output noise were drawn from the corresponding normal distributions.

To assess whether there were any group differences for each of the main four model parameters, we performed region of practical equivalence (ROPE) analysis using the respective hyperparameters. To this end, we determined for all retention and learning rates the posterior distribution of the difference between the hyperparameter reflecting the mean of the AD group and that of the healthy control group. We tested the ROPE for each parameter separately using the following formula (Kruschke, 2018):

(10) $$\mathrm{ROPE}=\pm 0.1\sqrt{(\sigma_{1}^{2}+\sigma_{2}^{2})/2},$$

in which $\sigma_{1}^{2}$ and $\sigma_{2}^{2}$ are the variances of the posterior distributions of the two groups. We accepted that there was no difference between groups if the 95% highest density interval (HDI) of the posterior distribution of the difference between groups fell completely within the ROPE. We rejected that there was no difference between groups if 95% HDI fell completely outside the ROPE. In all other cases, we withheld a decision.

### Data availability

Upon publication, all data and code will be available from the data repository of the Radboud University via the following URL: https://doi.org/10.34973/9x48-5d68.

## Results

In terms of neuropsychological characteristics, an independent sample *t* test on the IQ estimates (based on the NART) did not reveal any significant differences between the two groups (*t*_(27.5)_ = 2.0; *p *= 0.057; *t* test). However, as expected, the AD patients had worse global cognitive functioning than the control group (MoCA, *t*_(27.2)_ = 6.6; *p *< 0.001; *t* test) and worse declarative memory performance (doors test, *t*_(39)_ = 5.3; *p *< 0.001; MoCA-MIS, *t*_(28.4)_ = −10.3; *p *< 0.001; *t* test). Patients and controls were tested on making forward reaching movements to a visual target, using a protocol consisting of four blocks. A baseline phase of 100 trials was followed by a long phase of 240 trials during which they were exposed to a CW force field (except for the intervening ECs). This was followed by a shorter exposure of 30 trials to a CCW force field before the final EC block of 50 trials began.

### Force-field adaptation and spontaneous recovery in AD

**Figure 3.**
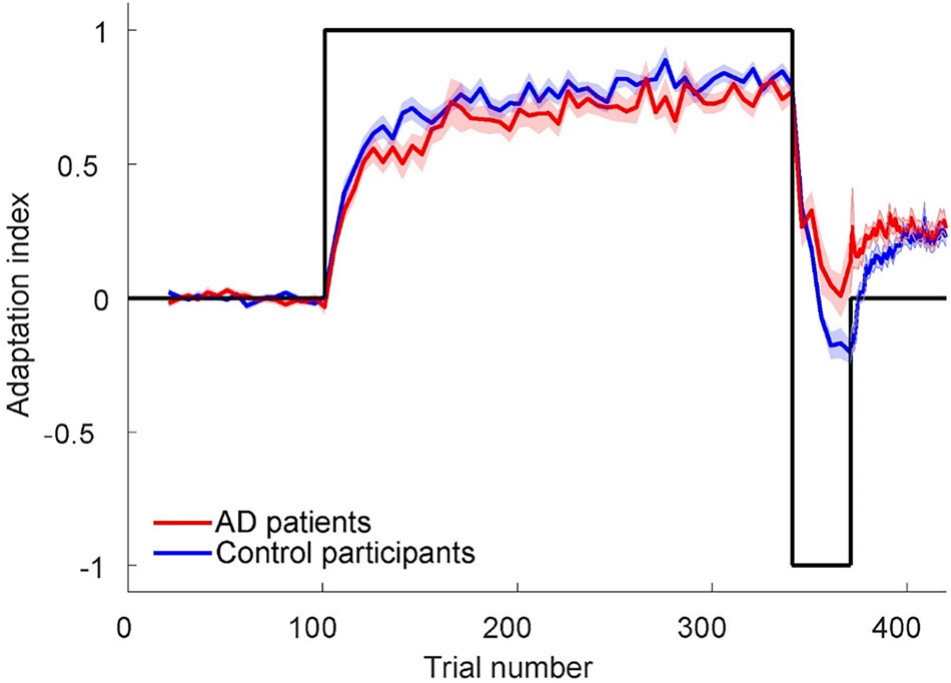
The average AI as a function of the trial number for control participants (blue) and AD patients (red). The black line indicates the direction of the force field (CW/CCW). The shaded area denotes ±SE.

To quantify adaptation within the first three blocks, every fifth trial was an EC trial (from trial 20 in the first baseline block), in which the robot clamped the reach to a straight line, while the compensatory force was measured, from which the AI was computed (see Materials and Methods). All trials in the fourth block were ECs. All patients and control participants who were included in the study were able to perform the task in accordance with the test instructions. Figure 3 shows the AI for AD patients and healthy controls during the four experimental blocks. As expected, force expression was unsystematic during the baseline phase since the robot did not perturb the reaches. In both groups, the AI gradually increased during exposure to the CW force field suggesting that like controls, patients also learned to compensate for the forces and approached an asymptotic level. While the asymptote was below one in both groups, indicating that neither patients nor controls completely cancelled the force applied by the robot, the patients compensated slightly but significantly less (*t*_(39)_ = −2.06; *p *= 0.046; *t* test). During the subsequent CCW block, when the force field had switched to the opposite direction, the AI quickly returned to baseline levels and for the controls even switched sign while starting to compensate for the CCW force field. This indicates that patients did not adapt as much to the second force field as the control participants (*t*_(39)_ = 2.97; *p *= 0.005; *t* test). Next, during the final EC block, the AI rapidly rebounded, in both patients and controls, expressing part of the compensatory strategy for the first force field, known as spontaneous recovery. The AI was initially larger in patients than in controls (*t*_(39)_ = 3.91; *p *< 0.0005; *t* test), but both groups plateaued at about the same level during the end of the EC phase. We next adopted a modeling approach to interpret this altered pattern of spontaneous recovery.

### The fast, not the slow adaptive process, is affected in AD

**Figure 4.**
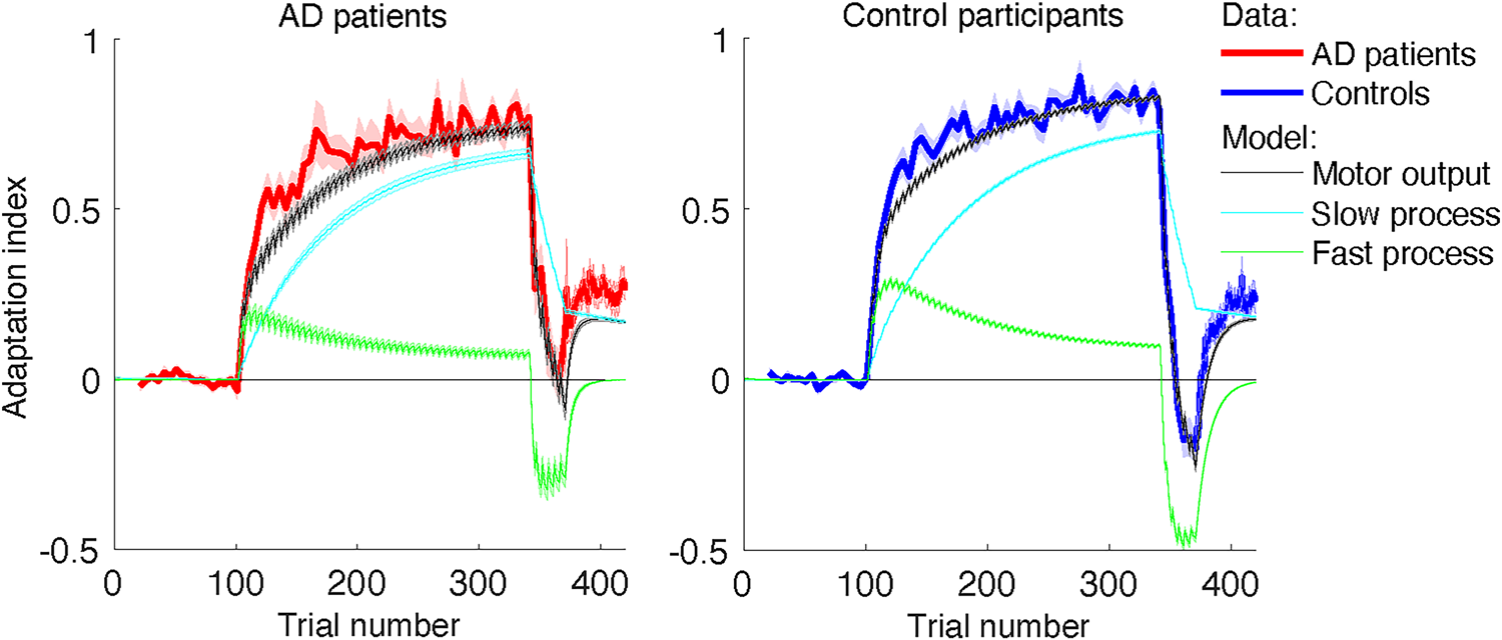
Model fit. AI as a function of trial number for the patients with AD (red; left panel) and control participants (blue; right panel). The fitted motor output (black) as a result of the slow process (cyan) and the fast process (green) is the mean of the posterior predictive check performed for individual subjects (500 random draws each). The shaded area denotes ±SE. The zigzag pattern in the model predictions during the adaptation phases arises from the succession of force-field trials (series of four trials during which adaptation increased) and error-clamp trials (single trials during which adaptation decayed). The data do not show the zigzag pattern because they show only the AI during the error-clamp trials (the only trials in which the AI was measured).

We fitted a Bayesian hierarchical version of a dual-rate adaptation model (M. A. Smith et al., 2006) to capture the time course of the AI. Figure 4 illustrates the two internal states of the model. The state of the fast process (in green) demonstrates quick learning and quick decay; the slow process (in light blue) illustrates slower learning and hardly decay. The model accounts for the main characteristics of the measured AI time courses, in both patients and controls. Upon closer inspection, however, there are subtle differences. First, both patients and controls generally adapt faster than the model implies. Second, the model does not fully capture the final level of spontaneous recovery. For both groups, the model shows lower spontaneous recovery than the data. Despite these subtle differences between data and model fits, the differences between the groups in the data are also visible in the posterior predictive checks of the model.

**Figure 5.**
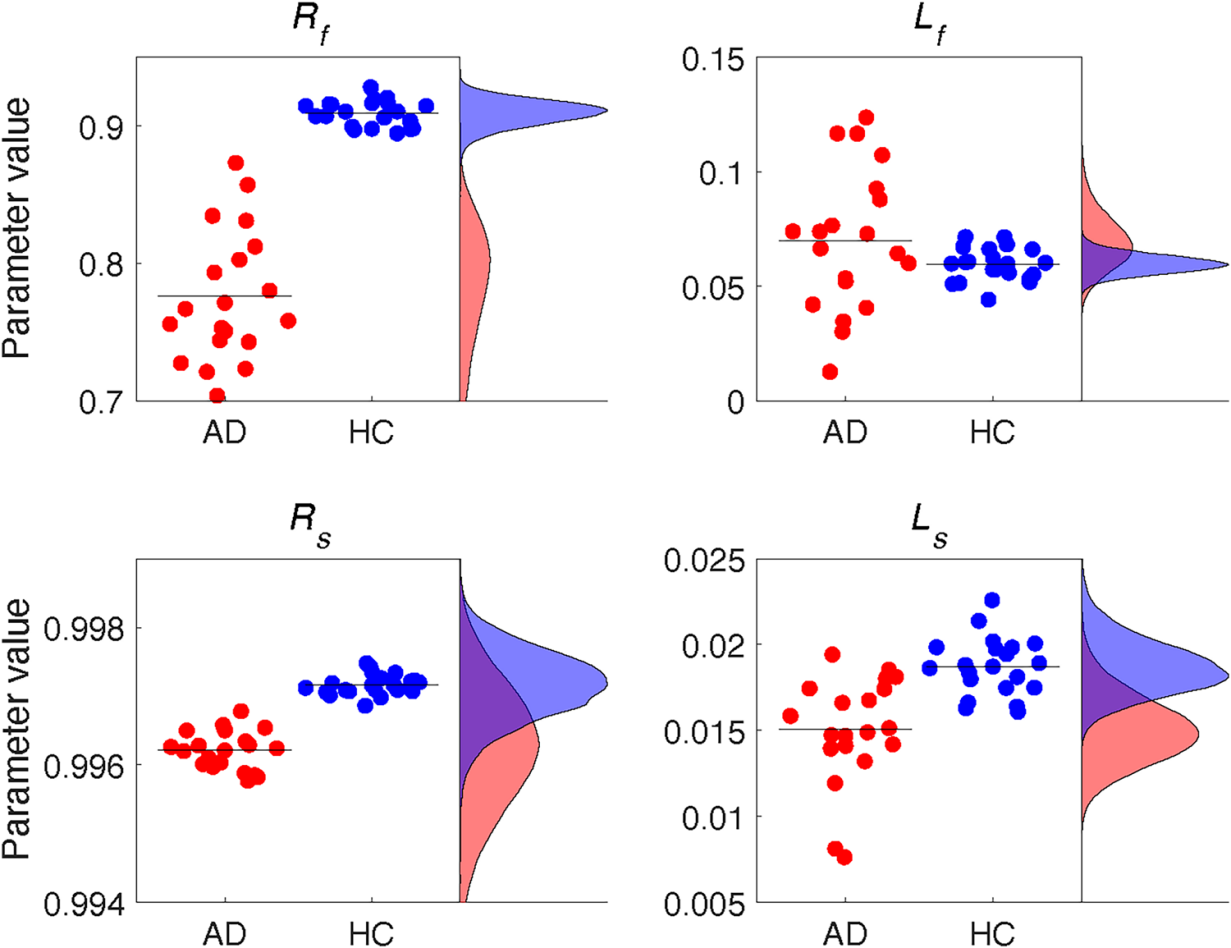
Individual parameter estimates (dots) and mean (black horizontal lines) and full posterior densities (on the right-hand side of each plot) of the hyperparameters representing the population mean of each parameter for AD patients (AD, red) and healthy control participants (HC, blue).

Figure 5 illustrates point estimates of the fitted parameters (based on the mean of the posterior distributions) of the hierarchical implementation of the two-state model for individual patients and controls, while the mean and the full posterior density of the hyperparameters representing the population mean of each parameter are also shown. We used a ROPE analysis to test whether there were differences in these hyperparameters between the groups. Consistent with our hypothesis, $R_{f}$ was the only parameter for which we can reject the null value: AD patients had a lower retention rate of the fast process than control participants (Fig. 5). For the other parameters ( $R_{s}$, $L_{s}$, and $L_{f}$), the ROPE fell completely inside the HDI of the posterior distributions of the parameters, indicating that the decision about these parameters is withheld. It should be emphasized that this also holds for the parameters of the slow process: the individual point estimates are samples that come from relatively broad densities of the hyperparameters. These results suggest that the differences in motor adaptation between the AD patients and controls can be explained by a reduced retention rate of the fast process in AD patients. We also used a ROPE analysis to test whether there were differences in these hyperparameters of the state and output noise between the groups, which was not the case for either noise parameter. Table 3 lists the means and the HDIs for all parameters.

**Table 3.** Mean and 90% HDI of the model parameters

Parameter	AD patients		Control participants	
	Mean	HDI	Mean	HDI
$L_{f}-\mu$	0.0700	0.0515–0.0922	0.0597	0.0530–0.0666
$L_{f}-\sigma$	0.0391	0.0254–0.0577	0.0114	0.0045–0.0185
$L_{s}-\mu$	0.0151	0.0120–0.0184	0.0187	0.0163–0.0216
$L_{s}-\sigma$	0.0051	0.0026–0.0081	0.0032	0.0004–0.0064
$R_{f}-\mu$	0.7765	0.6717–0.8453	0.9092	0.8927–0.9232
$R_{f}-\sigma$	0.0878	0.0269–0.1760	0.0175	0.0024–0.0357
$R_{s}-\mu$	0.9962	0.9946–0.9977	0.9972	0.9963–0.9980
$R_{s}-\sigma$	0.0010	0.0001–0.0024	0.0006	0.0001–0.0014
$\sigma_{\mathrm{state}}-\mu$	0.0343	0.0273–0.0420	0.0239	0.0184–0.0298
$\sigma_{\mathrm{state}}-\sigma$	0.0150	0.0081–0.0240	0.0124	0.0080–0.0178
$\sigma_{\mathrm{output}}-\mu$	0.1076	0.0834–0.1323	0.0942	0.0788–0.1092
$\sigma_{\mathrm{output}}-\sigma$	0.0624	0.0445–0.0857	0.0385	0.0281–0.0518
$L_{f}*$	0.0700	0.0407–0.1082	0.0597	0.0455–0.0746
$L_{s}*$	0.0150	0.0088–0.0218	0.0187	0.0141–0.0242
$R_{f}*$	0.7752	0.6060–0.8859	0.9092	0.8782–0.9347
$R_{s}*$	0.9962	0.9937–0.9983	0.9972	0.9958–0.9984
$\sigma_{\mathrm{state}}*$	0.0343	0.0207–0.0493	0.0239	0.0139–0.0353
$\sigma_{\mathrm{output}}*$	0.1077	0.0813–0.1328	0.0942	0.0747–0.1126

Parameter names in the table correspond to the parameters estimated by the model. For parameters that were estimated for individual participants (noted with an asterisk), the mean of the participants is listed.

**Figure 6.**
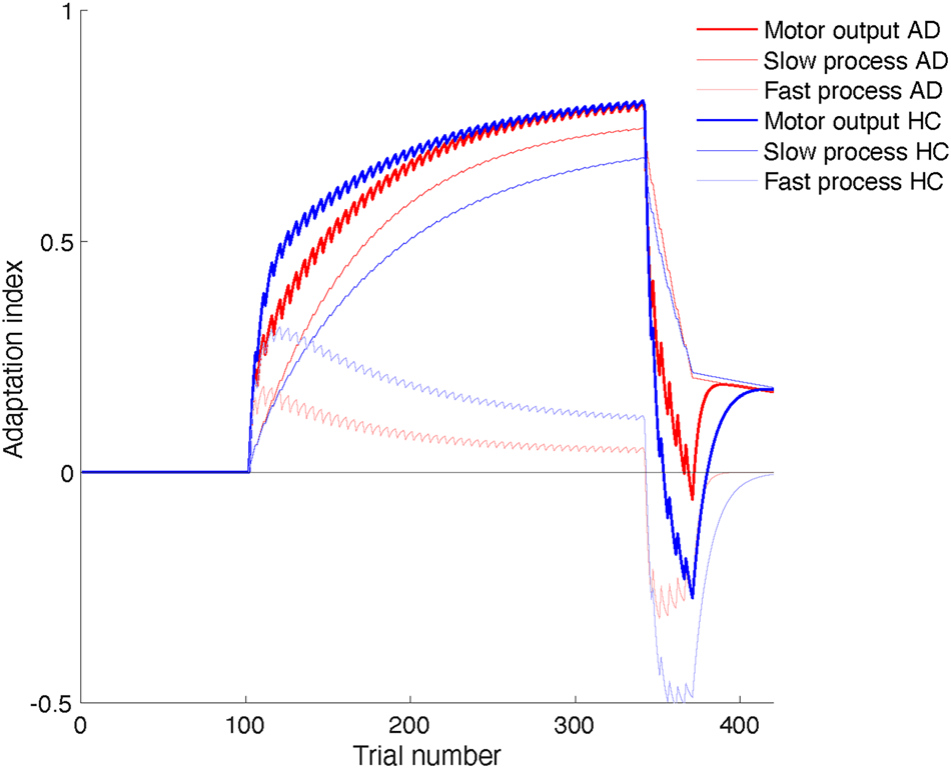
Simulation of the dual-rate model for two sets of parameters, representing AD patients and healthy control (HC) participants. For both groups: $L_{s}=0.0169$, $L_{f}=0.0649$, $R_{s}=0.9967;\;\;\mathrm{AD}\;\mathrm{patients},\;R_{f}=0.7765;\;\mathrm{HC},\;R_{f}=0.9092$. All noise levels were set to zero.

The conclusion that only the slow retention rate differed between the groups can fully explain the differences in the observed adaptation curves. Figure 6 shows simulations of the dual-rate model for two sets of parameters, representing AD patients and control participants. The parameters that did not differ between the groups were for both simulations set to the average estimate of both groups ( $L_{s}=0.0169$, $L_{f}=0.0649$, $R_{s}=0.9967$). The retention rate of the fast process was set to the mean of each experimental group ( $R_{f}=0.7765$ for the AD patients; $R_{f}=0.9092$ for the controls). The simulated controls adapt more rapidly to the first force field. This is because the fast process, which drives the initial adaptation, has better retention for this group than for the simulated patients. Another consequence is that the adaptation level of the fast process is lower for the simulated patients than for the controls, which in turn leads to stronger adaptation of the slow process for the simulated patients and almost the same net adaptation for both groups at the end of the adaptation phase. Due to their better retention, the fast process of the simulated controls adapted more strongly to the second force field than that of the simulated patients. Since the adaptation level of the slow process did not differ much between the groups, the net adaptation at the end of this phase was strongly negative for the simulated controls and close to zero for the simulated patients, consistent with the data. Finally, both simulated groups reached the same final level of spontaneous recovery, but the simulated patients reached this level earlier than the simulated controls, also consistent with the data.

## Discussion

We measured the time course of reach adaptation of early-stage AD patients, while they were exposed to perturbing force fields in the context of a spontaneous recovery paradigm. Guided by a dual-rate adaptation model, we reverse engineered the mechanisms underlying their motor learning ability in terms of a slow and a fast adaptive process.

Our results show that patients with declarative memory impairment compensated slightly less than healthy controls for the force perturbations, although both groups showed clear signs of spontaneous recovery. As M. A. Smith et al. (2006) demonstrated, effects of spontaneous recovery can be explained by the concurrent existence of multiple adaptive processes. This is confirmed by our dual-rate model analysis, which further shows that the fast, but not the slow, process is affected in AD. More specifically, the retention rate of this process—the recall of this state from trial to trial—was significantly lower than in controls, while the learning rate of the process, the error sensitivity, did not differ significantly.

In the motor learning literature, there is substantial behavioral evidence that explicit processes can contribute to motor adaptation (Heuer and Hegele, 2008; Anguera et al., 2010; Krakauer et al., 2019). Recent neuroimaging work corroborates these observations by demonstrating bidirectional interactions between neural substrates of motor memory and declarative memory (S. Kim, 2020), which change with age (Wolpe et al., 2020). The present findings constrain the role of the declarative memory to interactions with the fast adaptive process. This inference here is based on a clinical population with declarative memory impairment but concurs with findings in healthy participants using other paradigms. For instance, Keisler and Shadmehr (2010) demonstrated that a word-pair learning task, which taxes declarative memory, interferes with the memory of the fast, but not slow, process. Taylor et al. (2014) showed that explicitly declared performance reports during motor learning follow fast adaptation dynamics.

From a clinical perspective, it is commonly thought that patients with declarative memory deficits can learn some motor tasks, such as rotary pursuit, serial reaction time tasks, and mirror-tracking (Ferraro et al., 1993; Willingham et al., 1997; Dick et al., 2001; Rouleau et al., 2002; Hong et al., 2020; De Wit et al., 2022), because such tasks rely on implicit ways of learning. For example, Shadmehr et al. (1998) showed that an amnesic patient (H.M.), due to a resection of the medial–temporal lobe, was still able to adapt his reaches to a perturbing force field but was unaware that he learned the task. Noteworthy, the adaptation in this patient proceeded very slowly, as if only a slow process was involved and the fast adaptive process was lacking. Our memory-impaired patients still show evidence for the operation of a fast process, suggesting that the explicit processes are only partially affected. This may not be surprising given their mild declarative memory impairment, as indicated by lower MoCA-MIS and doors test scores in AD patients compared with those in the controls. Whether the contribution of the fast process would be smaller in AD patients at a more advanced disease stage remains to be investigated.

Recently, McDougle et al. (2022) described another case report of a patient (L.S.J.) with near-complete bilateral loss of her hippocampi. She was tested on various reach adaptation tasks, including a force-field paradigm for evoking spontaneous recovery. L.S.J. showed robust learning but weaker than controls, as well as spontaneous recovery, all consistent with the present observations. Furthermore, dual-rate modeling showed that L.S.J. differed from controls in the retention parameter $\left(R_{f}\right)$ of the fast motor learning process. The authors found this parameter to be increased, which they explain by increased rigidity of the fast process, reflecting reduced flexibility. In contrast, all our 20 AD patients showed a lower retention rate of the fast process compared with the controls, which can be expected assuming this parameter characterizes their amnesia. That being said, also the present study was not a randomized controlled trial, and interpretation should come with reservations in terms of generalizability and external validity.

We can only speculate about this difference with the study by McDougle and colleagues. Experimentally there seem to be only small differences (e.g., in the number and proportion of EC trials), but we tested a selective patient group (AD patients with MCI) which may be different than their individual case due to the neurodegenerative nature of AD. Furthermore, we used a hierarchical Bayesian modeling procedure to fit the dual-rate model, which is an advanced approach that deviates from McDougle et al.'s and other previous approaches (S. T. Albert and Shadmehr, 2018; Coltman et al., 2021). In this approach, the estimate of each of the four parameters ( $R_{s}$, $R_{f}$, $L_{s}$, and $L_{f}$) of each individual is simultaneously informed by the data from the other individuals, because all individuals inform higher-level parameters, called hyperparameters, which constrain all the individual parameters making them less sensitive to individual outliers (Kruschke, 2015). In other words, the hyperparameters constrain the estimates of the individual parameters of the retention and learning rates.

Based on an HDI + ROPE decision rule, the fast retention rate marked a significant neurocomputational alteration at the group level as well as at the level of each individual patient. For none of the other three parameters, this was found to be the case. Since a lower fast retention rate was robustly found in each patient, it opens up the possibility to use it as a prognostic or diagnostic marker to complement traditional taxonomies or neuropsychological test or to use it to track the development of the disease. The other parameters, including the learning rate, were not different between patients and controls. This suggests that sensitivity to error of the fast process is not changed in our patients, but rather what is remembered from trial to trial is affected. If such memory is lacking, one may not be able to form or enhance an explicit strategy that could contribute to the early phase of learning. Herzfeld et al. (2014) suggest that the error sensitivity depends on the history of past errors, which implies that the brain must store a memory of errors. Our results may suggest that this memory is affected in our patient group.

Other studies suggest that learning is constrained by the size of the error, rather than the sensitivity to error (Wei and Körding, 2009; H. E. Kim et al., 2018). The size of the error can be manipulated by the type of perturbation schedule that is used to elicit the adaptation. Using a gradual perturbation schedule in which participants never experience large errors, learning is suggested to be more implicit in nature (Orban de Xivry and Lefèvre, 2015) and may probe the slow process. Since the slow process learns only weakly from error, it may not depend, or depend less, on a memory of past errors, which would be consistent with the present results.

Our findings provide support for current disease management and clinical interventions that rely on the efficacy of errorless learning as an instructional method to facilitate learning in AD patients (Laffan et al., 2010; de Werd et al., 2013). There are two important types of error signals that drive sensorimotor adaptation: task performance error and the sensory prediction error. These errors are suggested to drive distinct learning processes, that is, sensory prediction errors drive an implicit learning, and task performance errors drive explicit learning strategies. In the clinic, errorless learning means that the patient is not given the opportunity to make a mistake, that is, makes no task performance errors. Using task-irrelevant clamped feedback paradigms, various studies have also shown that implicit adaptation occurs in the absence of task performance errors (Morehead et al., 2017; Poh and Taylor, 2019; Vandevoorde and Orban de Xivry, 2019). This is typically also what a gradual perturbation schedule in motor learning effectuates: while the participant never experiences a substantial task performance error, learning still takes place but without awareness and implicitly based on sensory prediction errors. So, errorless learning is likely an intervention that capitalizes on the slow adaptive processes in these patients, and as we show here, these processes are still intact in early AD patients. It is unknown whether further progression of the disease affects the slow adaptive processes as well. The cerebellum is argued to play a central role in learning from small errors (Criscimagna-Hemminger et al., 2010; Vandevoorde and Orban de Xivry, 2019) but is typically not regarded as a key region in the etiology of early-stage AD.

## Synthesis

Reviewing Editor: David Schoppik, New York University Decisions are customarily a result of the Reviewing Editor and the peer reviewers coming together and discussing their recommendations until a consensus is reached. When revisions are invited, a fact-based synthesis statement explaining their decision and outlining what is needed to prepare a revision will be listed below. The following reviewer(s) agreed to reveal their identity: NONE. Note: If this manuscript was transferred from JNeurosci and a decision was made to accept the manuscript without peer review, a brief statement to this effect will instead be what is listed below.

I've gone through the manuscript, the original reviews, and your rebuttal. By and large the revision addresses all of the concerns and is largely appropriate for publication in eNeuro. My only (strong) recommendation is that you change the title to a declarative statement rather than a question, so I'm returning this as "Revise - Reviewing Editor Review Only" instead of accepting it outright. Thank you for transferring the manuscript to eNeuro!

## Author Response

Dear editor, We hereby send the revised manuscript of the paper by Sutter and colleagues. The remaining comment we had to deal with was giving the title a more declarative nature. The manuscript is therefore now entitled: "Early-stage Alzheimer's disease affects fast but not slow adaptive processes in motor learning". We also added a link to the repository of the Radboud University where the readers can find the data and code of the study.

We hope that our manuscript will now be acceptable for your journal.

Sincerely, On behalf of all authors, Pieter Medendorp
